# Integrated single-cell and bulk RNA sequencing analysis identifies a cancer associated fibroblast-related signature for predicting prognosis and therapeutic responses in colorectal cancer

**DOI:** 10.1186/s12935-021-02252-9

**Published:** 2021-10-20

**Authors:** Hang Zheng, Heshu Liu, Yang Ge, Xin Wang

**Affiliations:** 1 Department of General Surgery, Peking University First Hospital, Peking University, Beijing, People’s Republic of China; 2 Department of Oncology, Beijing Chaoyang Hospital, Capital Medical University, Beijing, People’s Republic of China

**Keywords:** Single-cell RNA-seq, Colorectal cancer, Cancer-associated fibroblasts, Tumor microenvironment, Prognosis

## Abstract

**Background:**

Cancer-associated fibroblasts (CAFs) contribute notably to colorectal cancer (CRC) tumorigenesis, stiffness, angiogenesis, immunosuppression and metastasis, and could serve as a promising therapeutic target. Our purpose was to construct CAF-related prognostic signature for CRC.

**Methods:**

We performed bioinformatics analysis on single-cell transcriptome data derived from Gene Expression Omnibus (GEO) and identified 208 differentially expressed cell markers from fibroblasts cluster. Bulk gene expression data of CRC was obtained from The Cancer Genome Atlas (TCGA) and GEO databases. Univariate Cox regression and least absolute shrinkage operator (LASSO) analyses were performed on TCGA training cohort (n = 308) for model construction, and was validated in TCGA validation (n = 133), TCGA total (n = 441), GSE39582 (n = 470) and GSE17536 (n = 177) datasets. Microenvironment Cell Populations-counter (MCP-counter) and Estimate the Proportion of Immune and Cancer cells (EPIC) methods were applied to evaluated CAFs infiltrations from bulk gene expression data. Real-time polymerase chain reaction (qPCR) was performed in tissue microarrays containing 80 colon cancer samples to further validate the prognostic value of the CAF model. pRRophetic and Tumor Immune Dysfunction and Exclusion (TIDE) algorithms were utilized to predict chemosensitivity and immunotherapy response. Human Protein Atlas (HPA) databases and immunohistochemistry were used to evaluate the protein expressions.

**Results:**

A nine-gene prognostic CAF-related signature was established in training cohort. Kaplan–Meier survival analyses revealed patients with higher CAF risk scores were correlated with adverse prognosis in each cohort. MCP-counter and EPIC results consistently revealed CAFs infiltrations were significantly higher in high CAF risk group. Patients with higher CAF risk scores were more prone to not respond to immunotherapy, but were more sensitive to several conventional chemotherapeutics, suggesting a potential strategy of combining chemotherapy with anti-CAF therapy to improve the efficacy of current T-cell based immunotherapies. Univariate and multivariate Cox regression analyses verified the CAF model was as an independent prognostic indicator in predicting overall survival, and a CAF-based nomogram was then built for clinical utility in predicting prognosis of CRC.

**Conclusion:**

To conclude, the CAF-related signature could serve as a robust prognostic indicator in CRC, which provides novel genomics evidence for anti-CAF immunotherapeutic strategies.

**Supplementary Information:**

The online version contains supplementary material available at 10.1186/s12935-021-02252-9.

## Background

Colorectal cancer (CRC) is the most commonly diagnosed gastrointestinal cancer and is characterized by exhibiting cancer progression due to therapy resistance [1–3]. CRC predominantly originates from dysregulated epithelial cells as well as complex genetic abnormalities and molecular mechanisms [4, 5], and evolves by an accomplice called tumor microenvironment (TME), which comprises admixtures of stromal, immune and tumor cells as well as cytokines, chemokines and other extracellular matrix (ECM) acellular components [6]. The reciprocal and dynamic interactions between tumor cells and their surrounding TME play crucial roles in CRC tumorigenesis, progression and metastasis, as well as anticancer efficacy and drug resistance [7, 8], and have attracted wide attention in recent years.

As the major TME stromal cellular constituents, cancer-associated fibroblasts (CAFs) were found not only to promote tumorigenesis and enhance the aggressiveness of cancer cells, but also to induce chronic inflammation by producing pro-inflammatory cytokines that are responsible for immune tolerance and tumor metastasis [9–12]. Physiologically, quiescent fibroblasts are functionally activated into myofibroblasts in tissue remodeling conditions like wound healing and fibrosis processes, and are responsible for the integrity and equilibrium of ECM, which provides supportive framework for tissues. Once the remodeling process is completed, myofibroblasts are subsequently dwindling away through apoptosis [13]. Pathologically, CAFs are believed to be highly heterogeneous: genetic alterations in normal fibroblasts, pathological activation mediated by tumor cells [14], as well as additional transdifferentiation of epithelial and mesenchymal cells [15–17] have all been viewed as the origins of CAFs. In an interactional TME network inside the tumor, the “wound” does not heal [18] and the activated CAFs are resistant from apoptosis [19]. Excessive ECM proteins depositions and oncogenic molecules secreted constantly by CAFs are responsible for the invasion, stiffness, angiogenesis, immunosuppression, drug resistance and metastasis of CRCs [20–23]. Therefore, targeting CAFs along with current tumor cell-targeting agents could be promising therapeutic strategies to synergistically counteract CRC progression.

Understanding the heterogeneity and identifying the biomarkers of CAFs could be of great significance for survival prediction and CAF-based therapeutic guidance in CRC. Fibroblast activation protein (FAP), α-SMA (ACTA2), platelet derived growth factor receptor-β (PDGFRB), caveolin 1 (CAV1) and podoplanin (PDPN) are the generally recognized fibroblasts markers in CRC [20, 24–26]. Among them, FAP is the most appealing therapeutic target owing to its selective expressions in tumor as well as its unique collagenase and gelatinase activities [27, 28]. Preclinical studies have verified the promising effects of eliminating FAP-positive CAFs to increase the recruitments of anti-tumor CD8 + T cells into tumor stroma, hence rekindling the anti-tumor immunity and suppressing tumor progression [29, 30]. However, no further therapeutic efficacy was observed in metastatic CRC patients treated with anti-FAP inhibitor (sibrotuzumab, talabostat) in the subsequent clinical trials [31, 32]. Hence, more effort is needed for the investigations of innovative therapeutic targets to break the logjam of CAF-mediated tumor progression and immune suppression.

Recently, substantial interest has been aroused around advances in single-cell RNA sequencing (scRNA-seq) technology, which is capable of profiling genes as well as discovering distinct oncogenic cellular populations and associated markers at single-cell resolution [33]. Unequivocally characterizing tumor microenvironmental heterogeneity would facilitate uncovering drug resistance mechanisms and identifying more effective targets for individualized managements [34]. In this work, scRNA-seq profiles from Gene Expression Omnibus (GEO) (https://www.ncbi.nlm.nih.gov/geo/) colorectal cancer cells datasets were analyzed to describe the CAF subset and its marker genes. In addition, through integrated bioinformatics transcriptome analyses at both single-cell and bulk levels, we aimed to discover promising CAF-targeting therapeutic hallmarks and design a CAF-associated gene signature to predict drug sensitivity and prognosis for CRC patients.

## Methods

### Data source and preprocessing

The scRNA-seq files (raw unique molecular identifier (UMI) counts based on 10X Genomics technology) from five CRC tissues were accessed from GSE132257 [35] via GEO database (https://www.ncbi.nlm.nih.gov/geo/). We randomly picked four tissues (GSM3855011, GSM3855013, GSM3855017, GSM3855018) as the discovery cohort, and sample GSM3855015 was chosen for hallmark validation. The bulk transcriptome RNA-seq data and corresponding clinical data were obtained from The Cancer Genome Atlas rectal adenocarcinoma (TCGA‐READ) and colon adenocarcinoma (TCGA‐COAD) through the UCSC Xena browser (GDC hub) (https://gdc.xenahubs.net) [36]. The batch effects were modified through “ComBat” function of sva R package [37]. In total, 441 CRC samples with survival information were enrolled and then randomly assigned into TCGA training and TCGA validation cohort with a ratio of 7:3. Additionally, transcriptomic data of 470 CRC samples in GSE39582 and 177 CRC samples in GSE17536 were obtained as the external validation cohorts, expression values were respectively normalized via Robust Multi-array Average (RMA) algorithm, and genes mapped to multiple probes were summarized by their mean values.

### Single-cell RNA-seq analysis

We utilized Seurat R package (version 3.0.2) and applied standard downstream processing for scRNA-seq data (https://github.com/satijalab/seurat) [38]. Genes that detected in less than 3 cells as well as cells with less than 200 detected gene numbers were ruled out, and the mitochondria proportion was limited to less than 20%. Then, LogNormalize method was applied for data normalization. T-distributed stochastic neighbor embedding (t-SNE), a nonlinear dimensionality reduction method, was utilized after principal component analysis (PCA) for unsupervisedly clustering and unbiasedly visualizing cell populations on a two-dimensional map [39]. Subsequently, “FindAllMarkers” function was utilized to identify marker genes of each cluster with the filter value of absolute log2 fold change (FC) ≥ 1 and the minimum cell population fraction in either of the two populations was 0.25. In addition, the expression pattern of each marker gene among clusters were visualized by applying the “DotPlot” function in Seurat. Afterwards, SingleR package (version 1.0.0) was employed for marker-based cell-type annotation [40].

### Gene Ontology (GO) and the Kyoto Encyclopedia of Genes and Genomes (KEGG) Analyses

GO and KEGG pathway functional enrichment analyses were conducted through clusterProfiler R package (version 3.14.3) to assign various biological processes (BPs), molecular functions (MFs), cellular components (CCs) as well as pathways of identified marker genes in the interested cluster [41], P < 0.05 was regarded as statistically enriched.

### Construction and validation of an individualized CAF-related prognostic signature

We designed a prognostic signature across CRC patients by focusing on CAF marker genes, which were identified from the CAF-annotated scRNA-seq cluster. The main endpoint of this research was overall survival (OS), and prognostic CAF-related genes were investigated by univariate Cox regression model in the TCGA training dataset. Genes with P < 0.1 in univariate Cox analysis were regarded as candidate prognostic genes. To minimize overfitting risk, we then applied the least absolute shrinkage and selection operator (LASSO) Cox regression model via glmnet R package [42], and the CAF signature was calculated as: CAF risk score = Ʃ(β_i_ * Exp_i_), where β_i_ represented the LASSO coefficient of ith gene, and Exp_i_ was the ith candidate gene’s expression value. Subsequently, patients were classified into high- and low-CAF risk groups by their median CAF scores, and their relationship with OS was evaluated via Kaplan–Meier analysis. We generated heatmaps to visualize the association between CAF risk scores and candidate genes. Similarly, we validated our CAF signature in the TCGA validation and TCGA COAD/READ total cohorts, GSE39582 and GSE17536 external validation cohorts.

### Tumor microenvironment infiltration estimation

The relative infiltrations of 24 immune cell types were quantified via single sample gene set enrichment analysis (ssGSEA) with GSVA R package (version 1.34.0) [43]. The gene set for each immune cell subset was accessed from Bindea et al. [44]. Fibroblasts infiltration levels were quantified through Microenvironment Cell Populations-counter (MCP-counter) [45] and Estimate the Proportion of Immune and Cancer cells (EPIC) [46] algorithms via MCPcounter (version 1.2.0) [45] and immunedeconv (version 2.0.3) [47] R packages. Additionally, we applied estimate R package (version 1.0.13) to calculate the stromal and immune scores, which represents the tumor-associated stromal and immune infiltration levels of each sample [48].

### Chemotheraeutic sensitivity and immunotherapy response predictions

To predict chemosensitivity between high- and low-CAF risk groups, we used pRRophetic R package (version 0.5) to extrapolate half-maximal inhibitory concentration (IC50) values by building ridge regression model with ten-fold cross-validation [49, 50]. Several common anticancer drugs (camptothecin, docetaxel, gefitinib, gemcitabine, pazopanib, sunitinib) and their genetic profiles were obtained from the largest publicly accessible pharmacogenomics database: Genomics of Drug Sensitivity in Cancer (GDSC) (https://www.cancerrxgene.org/) [51]. In addition, Tumor Immune Dysfunction and Exclusion (TIDE) (http://tide.dfci.harvard.edu/) algorithm was implemented to predict immune checkpoint blockade therapy response between two groups [52].

### Gene set enrichment analysis (GSEA) in TCGA COAD/READ cohort.

To explore the different KEGG pathways and hallmark gene sets between high- and low-CAF risk groups, GSEA was performed with The Molecular Signatures Database (MSigDB) (c2.cp.kegg.v7.3.symbols, h.all.v7.3.symbols) via fgsea R package (version 1.12.0) [53, 54]. Pathways with an adjusted P < 0.05 were deemed to be significantly enriched.

### Nomogram construction and validation

Univariate and successive multivariate Cox regression analyses were performed to ascertain whether CAF model was independent of several clinical characteristics via survival R package. Subsequently, the nomogram was constructed based on the multivariate Cox regression coefficients of CAF signature and clinical variables in the TCGA training cohort. The concordance index (C-index) was calculated to validate the nomogram’s predictive performance, and calibration curves were also plotted to examine the consistence between the predicted 1-, 3- and 5-year OS probabilities and the actual observations (bootstrap-based 1000 iterations resampling validations).

### Human Protein Atlas (HPA) database and immunohistochemistry (IHC) verification

The protein expressions of these CAF signature genes in CRC tissues were analyzed in HPA online database (https://www.proteinatlas.org/), which aims to create a human proteome-wide map through integrated omics technologies [55].

For markers that are not available in HPA database, IHC will be further applied to examine the protein expression patterns in CRC samples who underwent radical rectal resection in Peking University First Hospital. The research was approved by ethics committee of Peking University First Hospital, and written informed consent was obtained from all subjects. Formalin-fixed paraffin-embedded surgical CRC specimens were collected and incubated with primary antibodies against CEBPD (sc-365546, Santa Cruz Biotechnology Inc., 1:200 dilution) and CXCL1 (ab89318, abcam, 1:200 dilution)) overnight at 4 °C, followed by incubation with secondary antibody (PV-9000, Beijing ZSGB-BIO) at room temperature for 30 min. Then, diaminobenzidine tetrachloride (DAB) staining (10 min at room temperature; ZLI-9019; Beijing ZSGB-BIO) was applied for CEBPD and CXCL1 expressions visualizations.

### Real-time polymerase chain reaction (qPCR)

Tissue microarrays containing 80 colon cancer samples (HColA095Su01) with the corresponding clinicopathological information were purchased from Shanghai Outdo Biotech (Shanghai, China) for prognostic verification. The mRNA expressions of the nine signature genes were quantified by qPCR using SYBR Green Premix (YEASEN, China) on the 7500 Sequence Detection System (Applied Biosystems, China). β-actin served as internal control for qPCR normalization, and the relative gene levels were calculated by 2^−ΔΔCT^ method. The primer sequences of these genes were listed in Additional file 1: Table S1.

### Statistical analysis

All statistical analyses and visualization were performed using R software v3.6.3 (https://www.r-project.org/). Wilcoxon test was applied for the comparisons between two groups. Survival analysis was performed using survival and survminer R packages. P-value less than 0.05 was regarded as statistical significance.

## Results

### Single-cell RNA-seq profiling, clustering and markers identifications

**Fig. 1:**
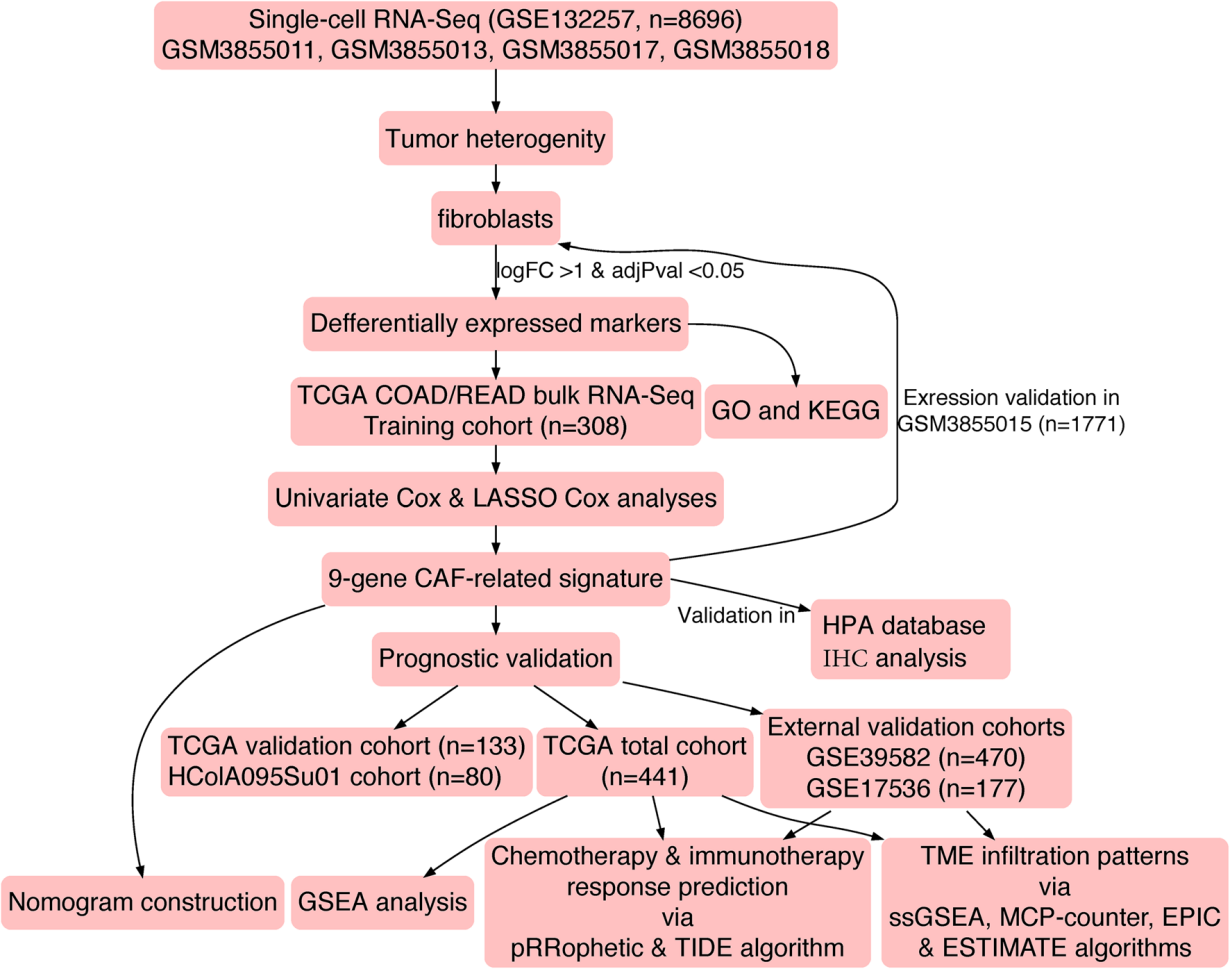
The schematic diagram of this study

**Fig. 2:**
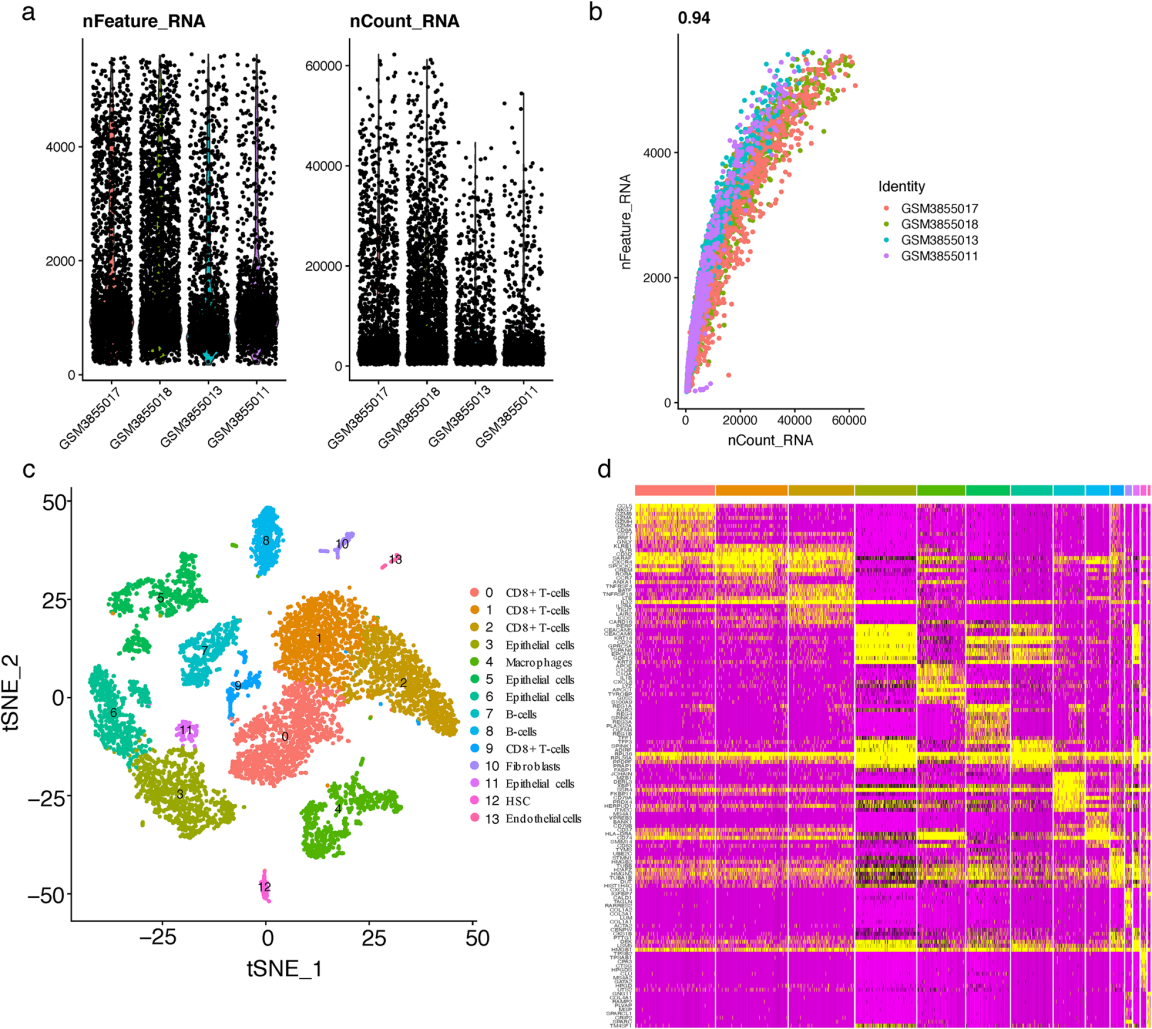
Analysis of single-cell RNA sequencing from 8696 cells of 4 CRC tissues. **a** Post quality control filtering of each sequenced cell, which was plotted in violin plots to display their number of RNA features (nFeature_RNA) and absolute UMI counts (nCount_RNA). **b** Correlation analysis between nFeature and nCount. **c** Cells were clustered into 14 types via tSNE dimensionality reduction algorithm, each color represented the annotated phenotype of each cluster. **d** Heatmap depicting expressions of top 10 marker genes among 14 detected CRC cell clusters

The overall study flow scheme was depicted in Fig. 1. After preprocessing scRNA-seq data based on the stringent quality control metrics as noted, 8696 high-quality cell samples isolated from the four discovery CRC tissues were screened and illustrated in Fig. 2a, and a strong positive correlation between numbers of detected genes (nFeature) and sequencing depth (total number of UMIs, nCount) was observed with the Pearson's correlation of 0.94 (Fig. 2b). We subsequently adopted t-SNE technique on the top 20 principal components to visualize the high dimensional scRNA-seq data, and successfully classified cells into fourteen subclasses, which were later annotated to acknowledged cell types using SingleR R package (Fig. 2c). The following major cell types were characterized: CD8 + T-cells, epithelial cells, macrophages, B-cells, fibroblasts, HSC and endothelial cells. In addition, the fibroblasts cluster was manually verified through well-acknowledged CAF markers (ACTA2, FAP, PDGFRB, CAV1, PDPN, PDGFRA, ZEB1, FOXF1, SPARC, MMP2, FN1) [25, 56], which further conformably confirmed the veracity of automated CAF cluster annotation (Fig. 6a, c). Furthermore, significant expressed marker genes across each group were identified with a threshold of logFC > 1 and adjPval < 0.05, and the top 10 significant differential markers of each cluster was displayed via heatmap (Fig. 2d).

Meanwhile, similar analyses were performed on the validation sample (GSM3855015), a total of 1771 high-quality cell samples were screened (Additional file 2: Fig. S1a), the Pearson's correlation between nFeature and nCount was 0.95 (Additional file 2: Fig. S1b). Eleven clusters were classified via t-SNE technique and annotated as six types of cells (CD8 + T-cells, epithelial cells, macrophages, B-cells, fibroblasts and HSC; Additional file 2: Fig. S1c), and the top 10 significant differential markers of each cluster was visualized in Supplementary Fig. 1d. Similarly, the fibroblasts cluster was manually authenticated through the above summarized CAF markers (Additional file 3: Fig. S2a, c).

### GO and KEGG functional downstream analyses of fibroblast marker genes

**Fig. 3:**
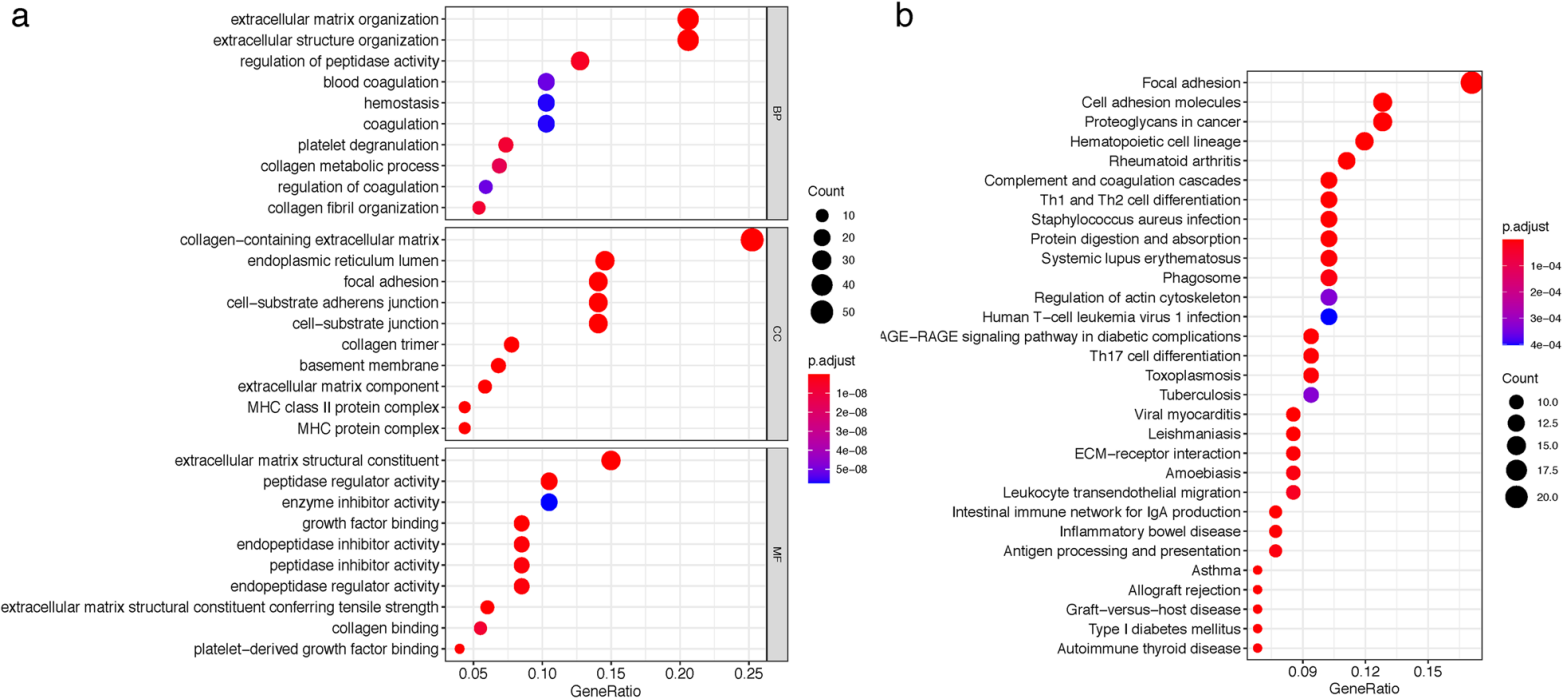
Bubble map of **a** the top 10 GO terms and **b** KEGG pathway enrichment analysis of 208 significant expressed marker genes in the fibroblasts cluster

We conducted GO and KEGG enrichment analyses to investigate the correlative functions and pathways of the above 208 genes in cluster 10 (fibroblast cluster). As shown in Fig. 3a, extracellular matrix organization, extracellular structure organization, collagen-containing extracellular matrix and extracellular matrix structural constituent were the main significantly enriched GO terms. Figure 3b exhibited the top 30 enriched KEGG pathways, which involved mainly in the desmoplastic and immune processes, such as focal adhesion, cell adhesion molecules, proteoglycans in cancer, Th1 and Th2 cell differentiation, antigen processing and presentation and leukocyte transendothelial migration pathways. These enrichment terms strengthened that the cluster was appropriately annotated and reliable for marker screening.

### Nine-gene prognostic CAF signature construction and verification

**Fig. 4:**
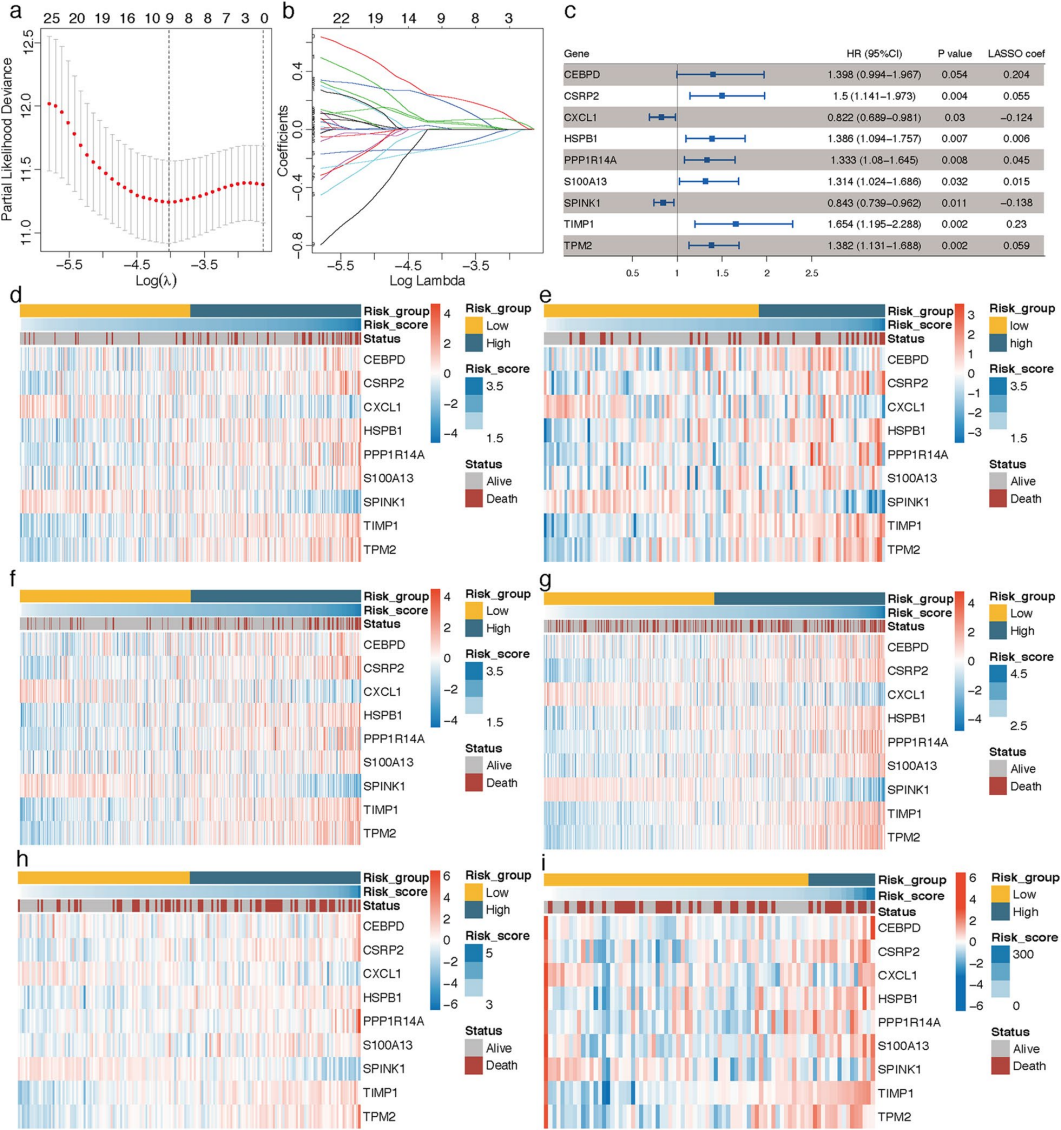
**a**, **b** LASSO Cox regression analysis identified 9 genes significantly correlated with overall survival in TCGA training cohort. (a) Ten-fold cross-validations for screening of the optimal parameter (lambda). **b** LASSO coefficient profiles determined by the optimal lambda. **c** Forest plot presented the HRs and P-values from the univariate Cox regression as well as LASSO coefficients of the nine prognostic signature genes. **d**–**i** Heatmap visualizing the expression levels of nine prognostic CAF genes with the CAF risk scores in **d** TCGA training, **e** TCGA validation, **f** TCGA overall, **g** GSE39582, **h** GSE17536 and **i** HColA095Su01 tissue microarray cohort

**Fig. 5:**
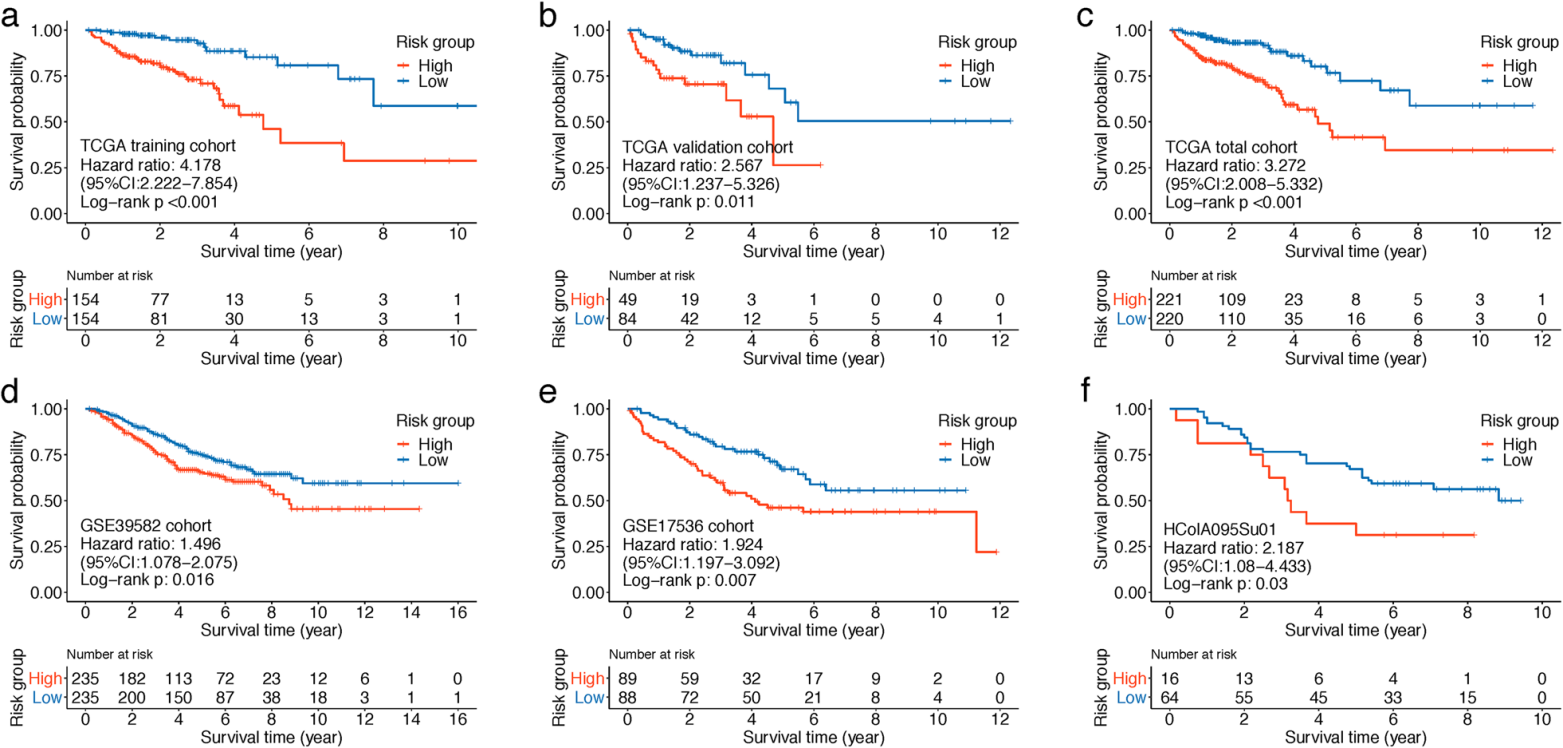
Kaplan–Meier curves displayed that high-CAF risk group had worse overall survival (OS) than low-CAF risk group in **a** TCGA COAD/READ training, **b** TCGA validation, **c** TCGA overall, **d** GSE39582, **e** GSE17536 and **f** HColA095Su01 tissue microarray cohorts

In the TCGA training cohort, by inputting the 208 differential CAF marker genes identified above into univariate Cox regression analysis, a total of 41 genes were exhibited with P < 0.1. LASSO Cox regression algorithm was then performed on these genes, the lambda.min was determined as the optimal lambda value by tenfold cross-validations, and 9 prognostic genes with non-zero coefficients were successfully identified (Fig. 4a, b). A nine‐gene CAF signature was subsequently constructed based on each gene’s expression level and its coefficient: risk score = (0.204341111 * expression of CEBPD) + (0.054785445 * expression of CSRP2) + (-0.123917745 * expression of CXCL1) + (0.005667789 * expression of HSPB1) + (0.044691324 * expression of PPP1R14A) + (0.014977193 * expression of S100A13) + (-0.137566435 * expression of SPINK1) + (0.230258936 * expression of TIMP1) + (0.058696341 * expression of TIMP2). Among the 9 prognostic genes, seven genes (HSPB1, S100A13, PPP1R14A, CSRP2, TPM2, CEBPD and TIMP1) were regarded as risk-related genes (HR > 1), while SPINK1 and CXCL1 were considered as protective genes (HR < 1) (Fig. 4c). Based on this risk formula, CAF risk score of each patient was calculated, and heatmaps illustrating the risk score and expression levels of the 9 genes in each cohort were displayed in Fig. 4d–i. Patients in TCGA training, TCGA total, GSE39582 and GSE17536 cohorts were divided into low- and high-CAF risk groups in the light of their median risk scores. The pairwise comparison of OS in different risk groups was investigated by log-rank test. Kaplan–Meier curves revealed that high CAF risk group had significantly unfavorable survival outcomes compared with the low CAF risk group (TCGA training cohort, hazard ratio (HR) = 4.178, 95% CI: 2.222–7.854, log-rank P < 0.001, Fig. 5a; TCGA total cohort, HR = 3.272, 95% CI: 2.008–5.332, log-rank P < 0.001, Fig. 5c; GSE39582 cohort, HR = 1.496, 95% CI: 1.078–2.075, log-rank P = 0.016, Fig. 5d; GSE17536 cohort, HR = 1.924, 95% CI: 1.197–3.092, log-rank P = 0.007, Fig. 5e). In TCGA validation and HColA095Su01 datasets, the optimal cutoff was determined by “sur_cutpoint” function of survminer R package, and higher CAF risk group patients also revealed worse OS than lower CAF risk group in TCGA validation (HR = 2.567, 95% CI: 1.237–5.326, log-rank P = 0.011, Fig. 5b) and HColA095Su01 (HR = 2.187, 95% CI: 1.08–4.433, log-rank P = 0.03, Fig. 5f) cohorts. In summary, this CAF model could serve as a reliable prognostic predictor for CRC patients.

**Fig. 6:**
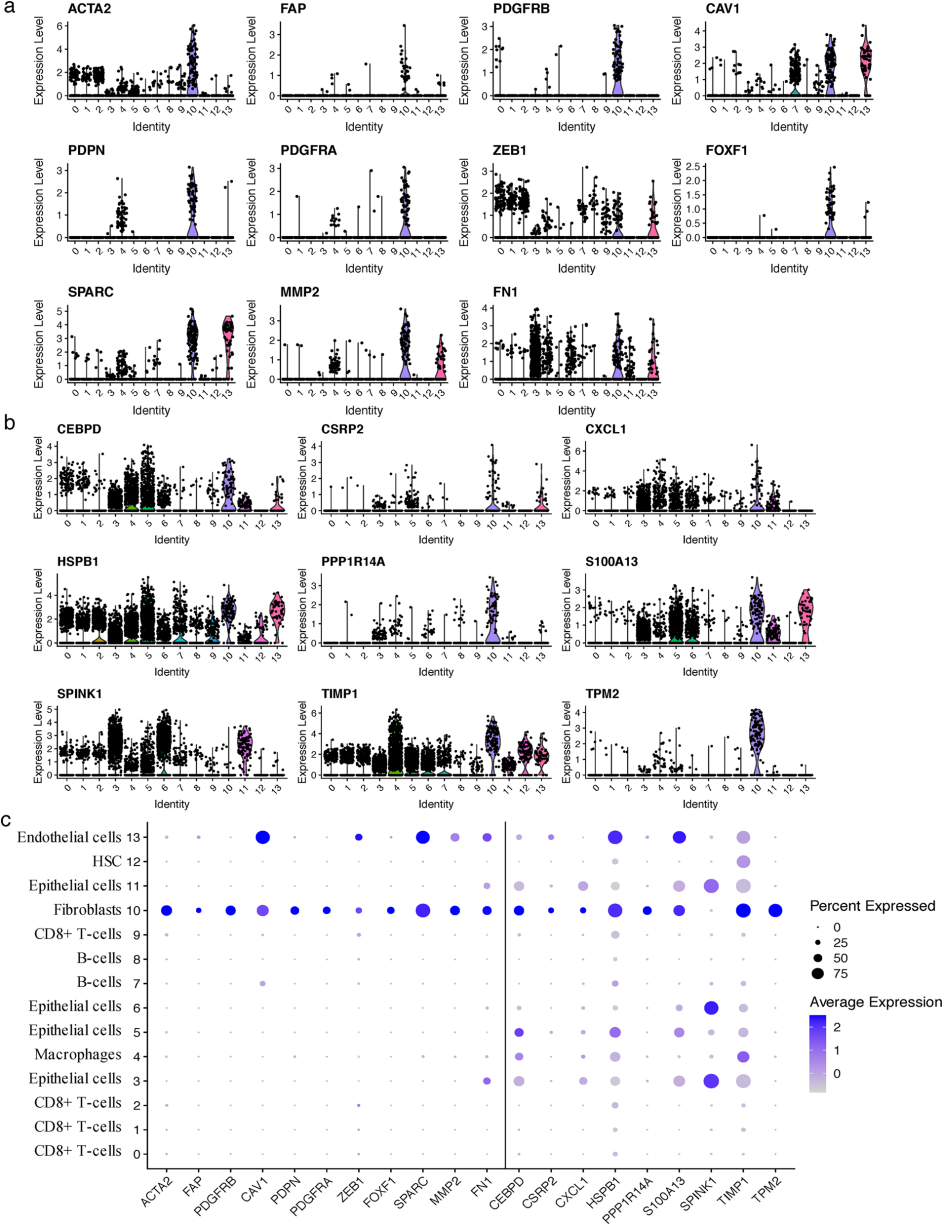
**a** Recognized and **b** the nine identified CAF markers expressions in CRC single-cell clusters. **c** Bubble plot visualizing genes expression characteristics in single-cell RNA sequencing profile. Cell phenotypes were listed on y-axis, recognized CAF markers (left part of the dotted line) as well as the identified nine prognostic markers (right part of the dotted line) were listed along the x-axis. Dot size reflects each gene’s expressing percentage of each cluster’s cells; dot color represents the expression level

Subsequently, expression characteristics of the nine markers among clusters in single-cell RNA sequencing profile were visualized via violin and bubble plots, CSRP2, PPP1R14A and TPM2 were up-expressed mainly in fibroblasts, while SPINK1 was down-regulated and expressed exclusively in epithelial cells. The other six markers were upregulated in fibroblasts cluster and revealed multiple expression forms among clusters (Fig. 6b, c). Moreover, similar results were reached through the external expression validation in GSM3855015, which confirmed the robustness of these genes as markers of CAFs (Additional file 3: Fig. S2b, c).

### TME infiltration patterns with CAF risk group

**Fig. 7:**
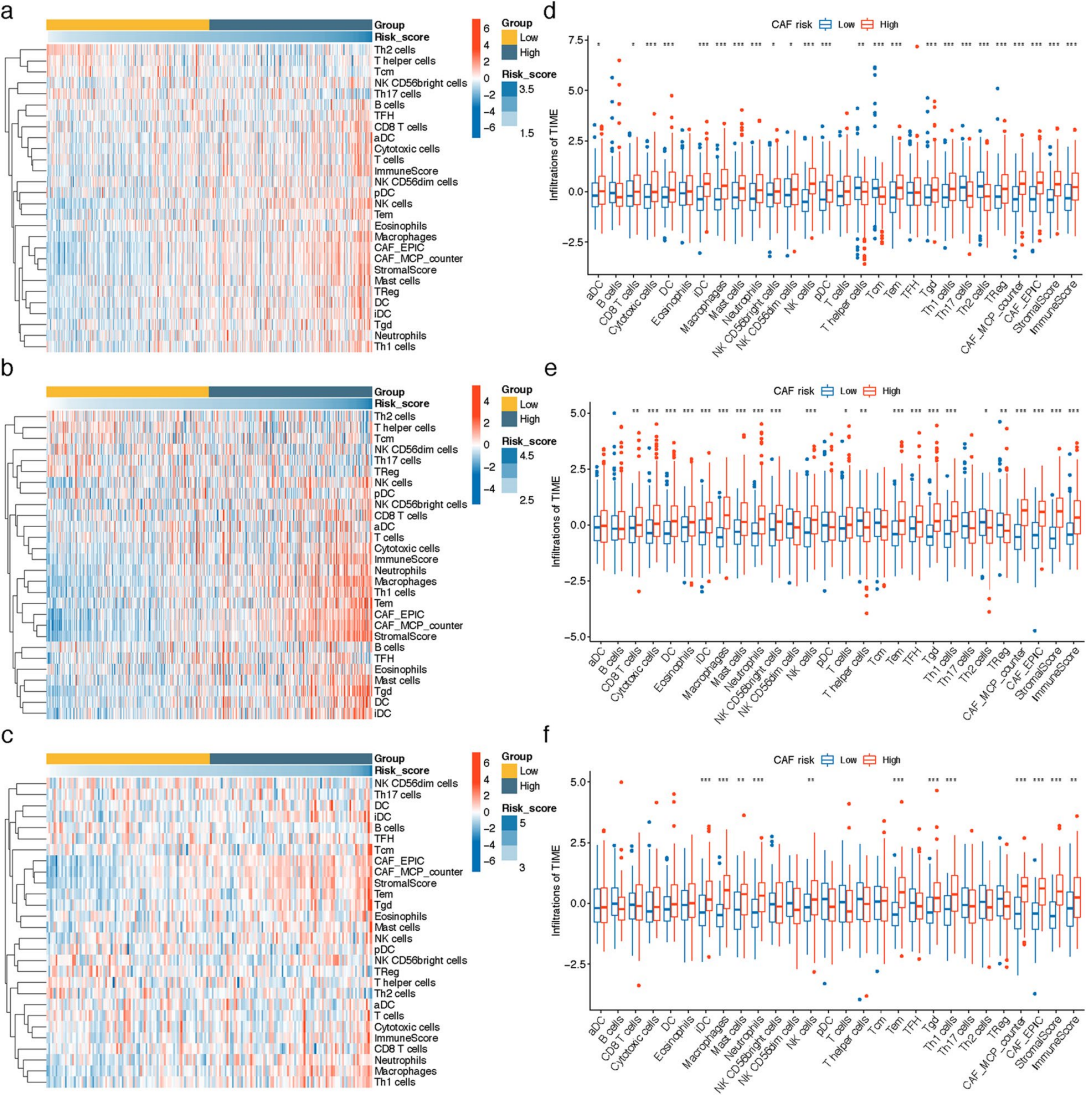
**a**–**c** Heatmap illustrating the distributions of 24 immune cell subsets, fibroblasts, stromal and immune scores assessed via ssGSEA, MCP-counter, EPIC and ESTIMATE algorithms in **a** TCGA COAD/READ, **b** GSE39582 and **c** GSE17536 cohort. (d-f) Wilcoxon analysis of the differing TME subtype distributions (z-score standardized) between high- and low-CAF risk groups in **d** TCGA COAD/READ, **e** GSE39582 and **f** GSE17536 cohort (* P < 0.05, ** P < 0.01, *** P < 0.001)

By running ssGSEA, MCP-counter, EPIC and ESTIMATE algorithms, we investigated the correlation of CAF risk score and TME constituents at bulk RNA-sequencing level. As shown in Fig. 7, pooled results of heatmaps and Wilcoxon analyses on TCGA COAD/READ, GSE39582 and GSE17536 datasets revealed the stromal and immune scores, as well as the infiltrations of several TME contents like fibroblasts, Th1 cells, Tgd, Tem, NK cells, neutrophils, Mast cells, Macrophages, iDC, cytotoxic cells and CD8 T cells were higher in high CAF risk group.

### CAF-related signature was predictive to chemotherapy and immunotherapy response

**Fig. 8:**
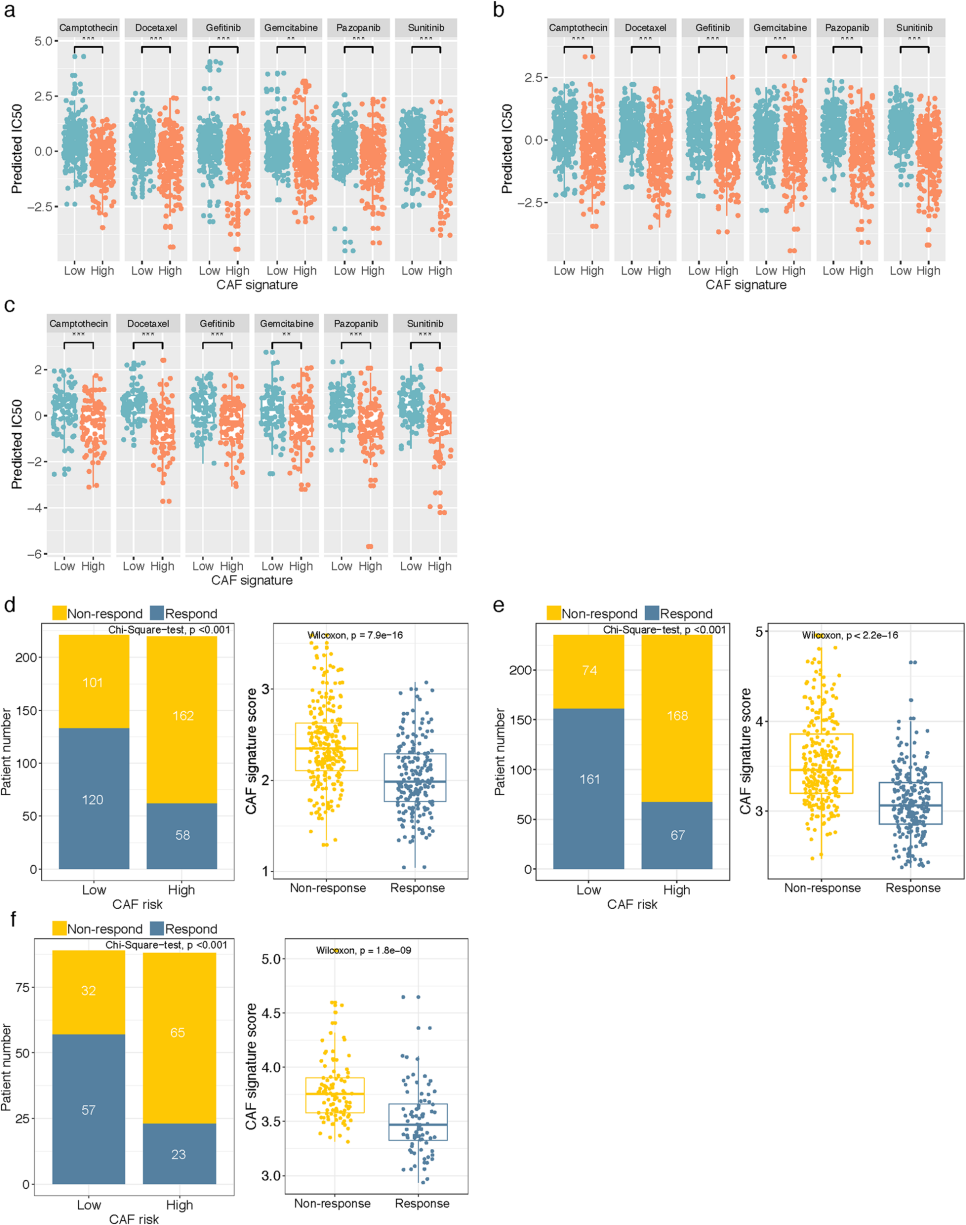
**a**–**c** The IC50 values of six anti-cancer drugs were predicted by pRRophetic algorithm and were Z-score normalized. The difference between high- and low-CAF risk groups of each drug in **a** TCGA COAD/READ, **b** GSE39582 and **c** GSE17536 cohort was compared by Wilcoxon test (** P < 0.01, *** P < 0.001). **d**–**f** TIDE analysis for predicting the likelihood of clinical response to immune checkpoint inhibitors in **d** TCGA COAD/READ, **e** GSE39582 and **f** GSE17536 cohort. Patients with a lower CAF risk score are more likely to respond from immune therapy

We next investigated the practicability of the model in guiding systemic therapies. Firstly, estimated IC50 values were calculated by pRRophetic algorithm to predict the different chemotherapy responses of CAF-associated high and low risk groups. Based on the GDSC cancer cell line database, we found that higher CAF risk score increased the sensitivity of 6 types of anticancer drugs (camptothecin, docetaxel, gefitinib, gemcitabine, pazopanib, sunitinib) in TCGA COAD/READ, GSE39582 and GSE17536 datasets (Wilcoxon test, all P < 0.01; Fig. 8a–c). Subsequently, using the TIDE online algorithm, we predicted the probability of response to immune checkpoint inhibitors in the three datasets. In the TCGA cohort, as shown in Fig. 8D, low-CAF risk patients (54.3%, 120/221) were more reactive to immunotherapy compared with high-CAF risk patients (26.36%, 58/220) (Chi-Square test, P < 0.001), in addition, responders presented markedly lower CAF scores compared to non-responders (Wilcoxon test, P < 0.001). Similarly, in the GSE39582 (Fig. 8e) and GSE17536 (Fig. 8f) validation cohorts, low-CAF risk group patients (68.51%, 161/235 in GSE39582; 64.05%, 57/89 in GSE17536) were also more responsive to immunotherapy than high-risk patients (28.51%, 67/235 in GSE39582; 26.14%, 23/88 in GSE17536) (Chi-Square test, both P < 0.001), and the responders presented significantly lower CAF scores in the two cohorts (Wilcoxon test, both P < 0.001). These results implied that while high CAF score was correlated with increased chemotherapy sensitivity, immunotherapy might be more effective in low CAF score CRC patients.

### Functional assessment of CAF signature

**Fig. 9:**
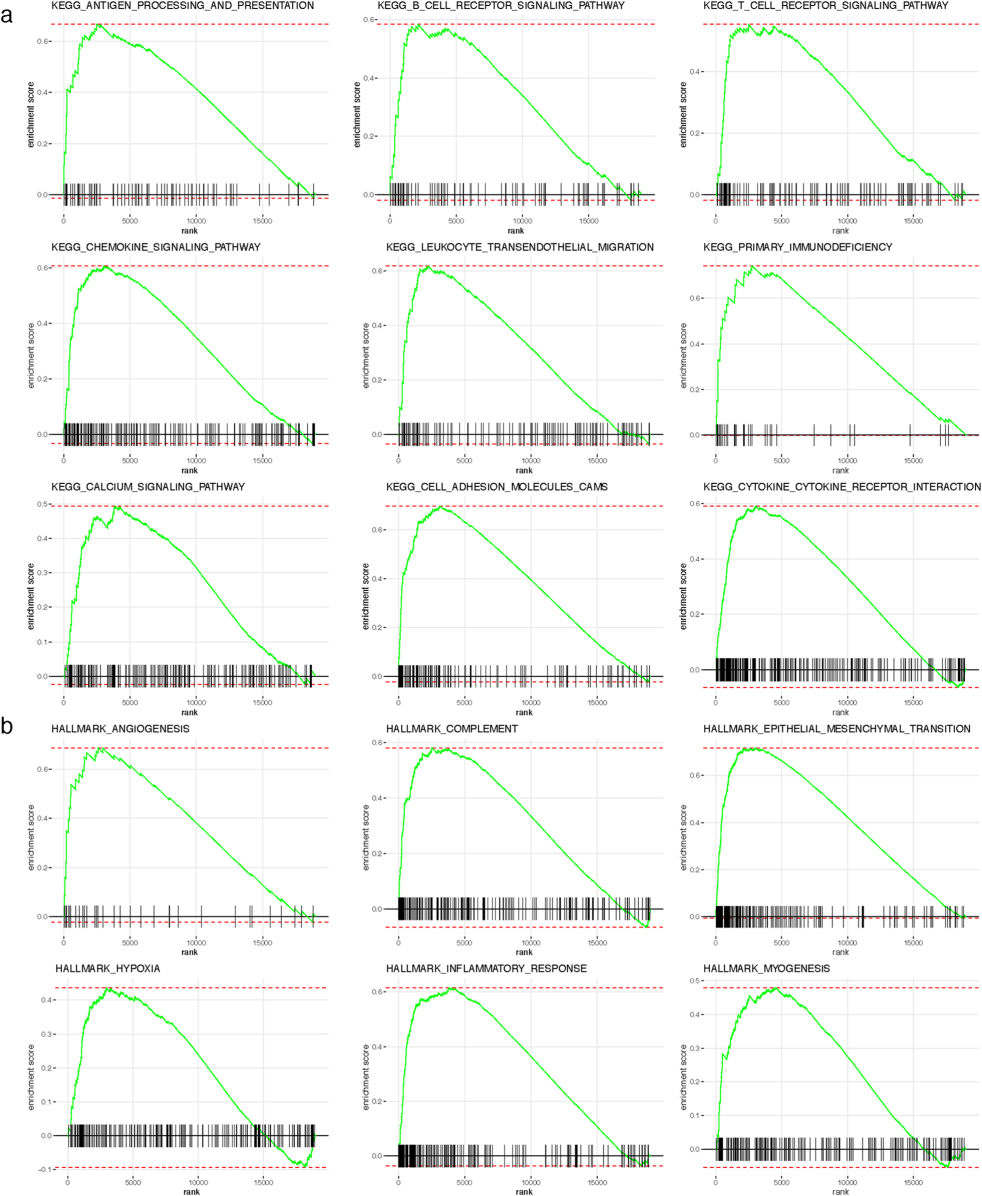
Gene Set Enrichment Analysis (GSEA) revealed the significant enrichment of **a** KEGG pathways and **b** hallmark genes sets in high CAF risk group patients compared with low CAF risk patients

Since the designed CAF signature was highly correlated with adverse prognosis and refractory immunotherapy response, we then investigated the functional pathways in this model through GSEA analysis. Patients in the TCGA COAD/READ cohort were separated into high- and low-risk groups based on their median CAF risk score as the cutoff value. We found several immune-related KEGG gene sets were enriched in high CAF risk group, including antigen processing and presentation, B cell receptor signaling pathway, T cell receptor signaling pathway, chemokine signaling pathway, leukocyte transendothelial migration, primary immunodeficiency, calcium signaling pathway, cell adhesion molecules cams, cytokine-cytokine receptor interaction (Fig. 9a). In addition, several hallmark gene sets, including angiogenesis, complement, epithelial mesenchymal transition, hypoxia, inflammatory response and myogenesis were also significantly enriched in high CAF risk group (Fig. 9b).

### CAF-related signature was an independent prognostic factor in CRC patients.

Univariate and multivariate Cox regression analyses were sequentially carried out in each cohort to examine whether the prognostic value of CAF-related gene signature was independent of other features including age and TNM stage. As shown in Table 1, in TCGA training, TCGA total, GSE39582, GSE17536 and HColA095Su01 cohorts, both univariate and multivariate Cox analysis manifested the CAF signature were independent prognostic factors. However, after adjusting for age and TNM stage parameters, the prognostic value of CAF signature was limited in TCGA validation cohort (HR = 1.334, 95% CI: 0.609–2.920, P = 0.471), we considered this was due in part to the relative small sample size in this cohort.

**Table 1:** Univariate and multivariate Cox proportional hazards regression analysis on OS

	Univariate analysis			Multivariate analysis		
	HR	95% CI	P	HR	95% CI	P
TCGA training cohort						
Age	1.023	0.999–1.049	0.06	1.033	1.008–1.058	0.009
TNM stage	2.67	1.918–3.718	< 0.001	2.73	1.911–3.9	< 0.001
CAF signature	4.418	2.521–7.744	< 0.001	3.161	1.828–5.468	< 0.001
TCGA validation cohort						
Age	1.061	1.024–1.099	0.001	1.058	1.023–1.094	0.001
TNM stage	2.363	1.566–3.564	< 0.001	2.391	1.566–3.651	< 0.001
CAF signature	2.323	1.094–4.932	0.028	1.334	0.609–2.920	0.471
TCGA total cohort						
Age	1.036	1.016–1.057	< 0.001	1.039	1.02–1.059	< 0.001
TNM stage	2.553	1.973–3.304	< 0.001	2.568	1.962–3.361	< 0.001
CAF signature	3.45	2.212–5.381	< 0.001	2.261	1.454–3.516	< 0.001
GSE39582 cohort						
Age	1.039	1.024–1.053	< 0.001	1.037	1.023–1.052	< 0.001
TNM stage	1.688	1.315–2.167	< 0.001	1.646	1.272–2.13	< 0.001
CAF signature	1.744	1.277–2.382	< 0.001	1.455	1.055–2.007	0.022
GSE17536 cohort						
Age	1.006	0.988–1.025	0.492	1.027	1.006–1.047	0.01
TNM stage	2.855	2.112–3.859	< 0.001	3.323	2.373–4.653	< 0.001
CAF signature	2.553	1.413–4.613	0.002	3.018	1.644–5.539	< 0.001
HColA095Su01 cohort						
Age	0.995	0.969–1.022	0.704	0.997	0.97–1.026	0.86
TNM stage	1.069	0.631–1.811	0.803	1.239	0.714–2.148	0.446
CAF signature	1.003	1.001–1.005	0.011	1.003	1.001–1.005	0.014

*OS* overall survival, *HR* hazard ratio, *CI* Confidence interval, *CAF* Cancer associated fibroblast

**Fig. 10:**
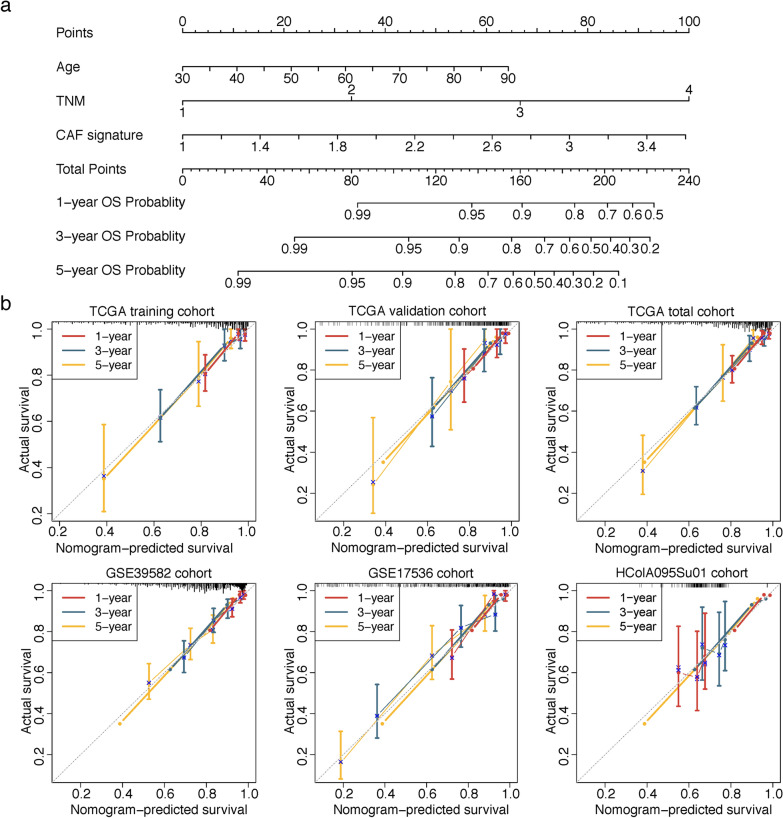
Nomogram construction and calibration plot validations. **a** Nomogram based on age, TNM stage and CAF signature for 1-, 3- and 5-year OS predictions. **b** Calibration curves for testing the agreement between 1-, 3- and 5-year predicted overall survival and actual observations in TCGA training, TCGA validation, TCGA entire, GSE39582, GSE17536 and HColA095Su01 cohorts

Based on the multivariate Cox regression coefficients of nine-CAF-related gene signature and clinical traits (age and TNM stage) in the TCGA training cohort, we built a prognostic nomogram for clinicians to quantitatively predict 1, 3 and 5-year OS probabilities of CRC patients (Fig. 10a). The C-index of the nomogram was 0.813 (95% CI = 0.746–0.88) in TCGA training set, 0.772 (95% CI = 0.677–0.867) in TCGA validation set, 0.792 (95% CI = 0.736–0.848) in TCGA total set, 0.658 (95% CI = 0.611–0.705) in GSE39582 set, and 0.78 (95% CI = 0.726–0.834) in GSE17536 set. The calibration curves manifested the model’s predictions of 1-, 3- and 5-year OS probabilities were favorably consistent with the ideal predictions (gray line) in all datasets (Fig. 10b). These results demonstrated the aggregated nomogram model could serve as a reliable tool for OS predictions of CRC patients.

### Study of the expression patterns of CAF-related signature genes at protein levels via HPA database and IHC analyses

**Fig. 11:**
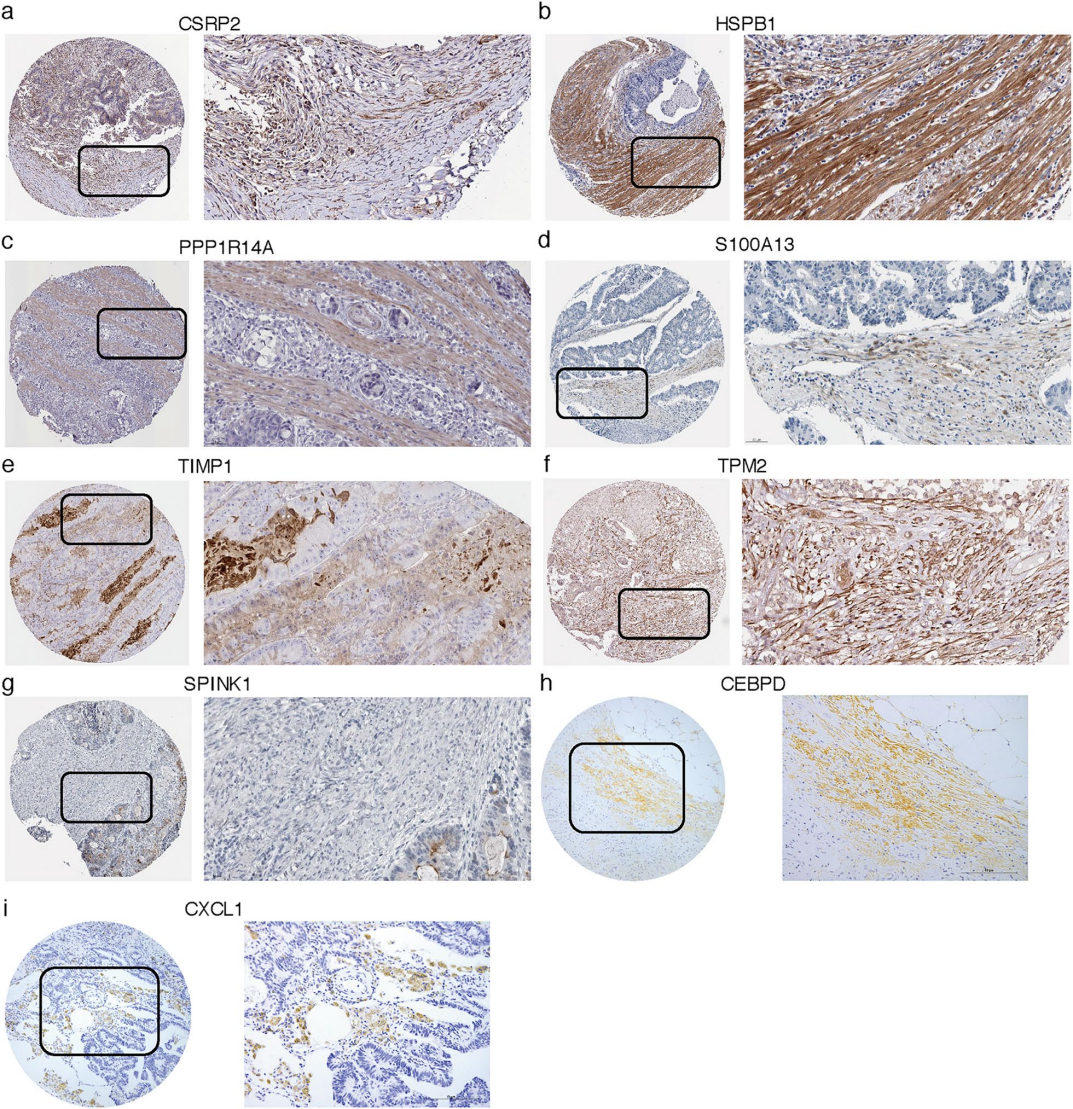
(a-g) Immunohistochemistry showing the protein expressions of **a** CSRP2, **b** HSPB1, **c** PPP1R14A, **d** S100A13, **e** TIMP1, **f** TPM2 and **g** SPINK1 based on the Human Protein Atlas (HPA) database. **h**, **i** Immunohistochemistry images of **h** CEBPD and **i** CXCL1 protein expressions in CRC tissues

Finally, with respect to CRC tissue protein expression levels, the immunohistochemical results from HPA database indicated that protein expression of CSRP2, HSPB1, PPP1R14A, S100A13, TIMP1 and TPM2 was higher in CRC stroma (Fig. 11a–f), while SPINK1 was weakly expressed in interstitial areas (Fig. 11g), and there was no immunohistochemical data for the other 2 genes (CEBPD and CXCL1) in HPA database. Hence, IHC analyses were performed on these two genes, and the examples of IHC staining of CEBPD and CXCL1 were shown in Fig. 11h, i. The expressions of CEBPD (Fig. 11h) and CXCL1 (Fig. 11i) were also found mainly in stromal spaces of CRC tissues.

## Discussion

The recognized tumor-promoting capacity of CAFs makes them a prospective immunotherapy target [9, 11, 57]. However, the clinical applications remain challenging owing to the lack of effective targetable biomarker, which motivated us to investigate the novel CAF markers in CRC. In this study, by analyzing single-cell genome of the GEO CRC patient datasets, we clearly revealed the fibroblasts subset and characterized 208 fibroblast markers with highly altered expressions that could not be discriminated in bulk RNA-seq. Since CAFs revealed a high level of heterogeneity [58], a model composed of multiple biomarkers would achieve an improved prognostic efficacy over the individual biomarker. Therefore, combined with the integrated analysis of TCGA COAD/READ, GSE39582 and GSE17536 bulk sequencing projects as well as HColA095Su01 experimental verification cohort, we for the first time established and validated a robust nine-gene CAF-related molecular signature capable of predicting prognosis, estimating TME stromal and fibroblasts components and therapy response for CRC patients. Univariate and multivariate Cox regression analyses verified the CAF-related signature was an independent risk factor associated with OS. To improve the predictive efficacy of the signature and facilitate clinical application, we subsequently constructed and validated a nomogram based on age, TNM stage and CAF signature for clinical practicality to predict OS.

In this study, we observed the immune and stromal cells infiltrations were more abundant in the high CAF risk group, especially several immunosuppressive types like fibroblasts, macrophages and mast cells. The effectiveness of most immunotherapies depends on the abundant infiltrations of CD8 + T cells in the tumors [59]. However, while high CAF risk group harbored relative richer infiltrations of Th1 cells, cytotoxic cells and CD8 T cells, TIDE algorithm manifested that high CAF risk CRC patients were more likely not to respond to anti-PD-1 and anti-CTLA-4 therapies in all three datasets. This could be partly explained by the findings from Ford et al. that CAFs modulate immunotherapies resistance specifically by discharging CD8 + T cells from tumor mass to tumor margin [60], meanwhile, the remodeling ECM constructed interactively by CAFs and cancer cells serve as the physical hamper against the penetration of tumoricidal immune cells as well as the delivery of anticancer agents to solid tumors [20, 61]. Interestingly, pRRophetic algorithm indicated that high-CAF risk CRC patients were more sensitive to several conventional chemotherapy agents than low-CAF risk patients. Researchers have demonstrated the anti-tumor immune efficacy of traditional chemotherapeutics [62]. For example, McDonnell et al. reported the standard-dose gemcitabine would enhance the tumor-associated antigens cross‐presentation efficacy of tumor‐resident dendritic cells to enable the reactivation capacity of tumor‐infiltrating CD8 + T cells [63]. We proposed a promising therapeutic strategy of combining conventional chemotherapy, natural-based substitutes [64, 65] and CAF-targeting immunotherapy to stimulate intratumoral CD8 + T-cell penetrations and resensitize high CAF-risk tumors to the current T cell based immunotherapies. However, further studies are needed for the design of synergistic therapies.

For the CAF-related genes in this well-established signature, scRNA-seq displayed TIMP1 expressed mostly in fibroblasts, epithelial cells, macrophages and endothelial cells. Many studies have identified dysregulation of TIMP1 expressions contributed critically to pro-tumor inflammation initiation, matrix remodeling and fibrosis development [66–68]. Illemann et al. reported that TIMP1 was generally expressed in α‐SMA‐positive myofibroblasts in both primary CRC and liver metastases, and promotes the anti-apoptosis and pro‐angiogenesis activities [69]. TIMP1 has also been verified to promote intra-tumoral CAFs infiltration, proliferation and migration by activating ERK1/2 kinase in CAFs [70]. In addition, upregulated TIMP1 in the cancerous CAF stroma would participate the vascular remodeling process and enhance the invasions of colorectal cancer cells [71]. Moreover, knockdown of TIMP1 could promote apoptosis of colon cancer cells via BCL2-Associated Agonist Of Cell Death (BAD) mediated phosphoration pathway and suppress the migration and invasion of cancer cells through downregulating Fibronectin and upregulating E-cadherin [72]. As the only downregulated gene in fibroblasts cluster, SPINK1 expressed mainly in epithelial cells and was regarded as a protective marker, which was consistent with previous studies [73–75]. However, in vitro experiments exhibited that SPINK1 contributed significantly to proliferation and invasion of CRC cell lines [76, 77]. This discrepancy could be partly explained by the concurrent expressions of SPINK1 and EGFR, which exerts distinct functions in CRC tissue [74]. The inconsistency also occurred in CXCL1, except for one study that demonstrated CXCL1 was a protective gene [78], other studies demonstrated that high CXCL1 expression in CRC epithelium correlated with adverse clinicalpathological characteristics and poor prognosis [79, 80]. CXCL1 is an inflammatory chemokine secreted mainly by CRC epithelia and myofibroblasts and is capable of driving tumor initiation and progression [81]. More experiments are needed to further validate the prognostic value of CXCL1 in CRC patients. Our results displayed PPP1R14A, TPM2 and CSRP2 were mainly expressed in fibroblasts cluster. PPP1R14A mRNA codes protein CPI-17, which directly deactivates tumor suppressor merlin (encoded by neurofibromatosis type 2 gene NF2) through merlin phosphorylation and tumorigenic Ras signaling activation [82, 83], and PPP1R14A has been reported to be aberrantly methylated in CRC [84] and serves as an epigenetic biomarker for CRC early detection [85]. A recent single-cell sequencing analysis from Zhou et al. also identified TPM2 as fibroblast-specific marker associated unfavorable prognosis in CRC [86]. Furthermore, Wang et al. reported the knockdown of CSRP2 would lead to the transformation from fibroblasts into CAFs and enhance their proliferation and migration capacities in gastric cancer [87], while its function in CRC fibroblasts still remains unclear. We showed CEBPD expressed mainly in epithelial cells, macrophages and fibroblasts. Chi et al. reported that CEBPD activation in M2 macrophages and myofibroblasts/CAFs led to the acquisition of chemoresistance and significantly promoted sphere-forming ability, stemness, invasion and metastasis in both responsive and drug-resistant breast cancers [88]. Wang et al. found CEBPD participated the integration of EMT and lipid metabolism signaling to promote lung adenocarcinoma metastasis [89]. S100A13 is a calcium binding protein gene [90], the digenic mutations of S100A13 would break calcium homeostasis, distort ECM and result in progression of lung fibrosis [91]. In addition, S100A13 is regarded as an angiogenic and prognostic biomarker in melanoma [92] and astrocytic gliomas [93]. Lee et al. demonstrated that HSPB1 is secreted from endothelial cells and physiologically modulates the balance of angiogenesis through interacting with vascular endothelial growth factor (VEGF). During the tumor-induced angiogenesis process, however, VEGF is overwhelmingly increased, yet the concomitant increase of HSPB1 is incapable of balancing the pathological angiogenesis, therefore contributes to tumor progression [94]. Nevertheless, the effects of CEBPD, S100A13 and HSPB1 on fibroblast-induced tumorigenesis in CRC have never been elucidated, which necessitate further investigations.

Several limitations in this study should be acknowledged. First, it was a retrospective study based on public sequencing data, and the sample capacity of the tissue microarray verification cohort was insufficient. Hence, the prognostic and predictive efficacies of our CAF-related signature should be prospectively verified in large clinical trial. In addition, cross-validations at proteomics level are also necessary to serve the clinical applications. Secondly, the molecular mechanisms of how these CAF-related genes affect patient prognosis and therapeutic responses need to be clarified by further basic experimental studies.

## Conclusions

In summary, based on integrated single-cell and bulk RNA sequencing analysis, we constructed and validated a nine-gene CAF-related signature as an independent prognostic indicator for CRC patients. Our results also provided genomic evidence for future research directions on anti-CAF therapeutic strategies for those who might not benefit from immunotherapy.

## Supplementary Information

**Additional file 1: Table 1**. qRT-PCR primer sequences.

**Additional file 2: Figure S1**. Validation analysis on single-cell RNA sequencing from 1771 cells of one CRC tissue (GSM3855015). (a) Post quality control filtering of each sequenced cell, which was plotted in violin plots to display their number of RNA features (nFeature_RNA) and absolute UMI counts (nCount_RNA). (b) Correlation analysis between nFeature and nCount. (c) Cells were clustered into 11 types via tSNE dimensionality reduction algorithm, each color represented the annotated phenotype of each cluster. (d) Heatmap depicting expressions of top 10 marker genes among 11 identified CRC cell clusters.

**Additional file 3: Figure S2**. (a) Recognized and (b) the identified CAF markers expressions in single-cell clusters of GSM3855015 sample. (c) Bubble plot visualizing genes expression characteristics in single-cell RNA sequencing profile. Cell phenotypes were listed on y-axis, recognized CAF markers (left part of the dotted line) as well as the identified 9 prognostic markers (right part of the dotted line) were listed along the x-axis. Dot size reflects each gene’s expressing percentage of each cluster’s cells; dot color represents the expression level.

## Data Availability

The datasets supporting the findings of this study are available in the from The Cancer Genome Atlas (TCGA) (https://gdc.xenahubs.net) and GEO (https://www.ncbi.nlm.nih.gov/geo/) databases. Further inquiries can be directed to the corresponding author.

## Abbreviations

CAF: Cancer-associated fibroblast
CRC: Colorectal cancer
scRNA-seq: Single-cell RNA sequencing
GEO: Gene Expression Omnibus
TCGA: The Cancer Genome Atlas
GDC: Genomic Data Commons
TME: Tumor microenvironment
ECM: Extracellular matrix
FAP: Fibroblast activation protein
ACTA2: Actin alpha 2
PDGFRB: Platelet derived growth factor receptor-β
CAV1: Caveolin 1
PDPN: Podoplanin
UMI: Unique molecular identifier
RMA: Robust Multi-array Average
READ: Rectal adenocarcinoma
COAD: Colon adenocarcinoma
t-SNE: T-distributed stochastic neighbor embedding
PCA: Principal component analysis
FC: Fold change
GO: Gene Ontology
KEGG: Kyoto Encyclopedia of Genes and Genomes
BP: Biological process
MF: Molecular function
CC: Cellular components
LASSO: Least absolute shrinkage operator
ssGSEA: Single sample gene set enrichment analysis
MCP-counter: Microenvironment Cell Populations-counter
EPIC: Estimate the Proportion of Immune and Cancer cells
IC50: Half-maximal inhibitory concentration
GDSC: Genomics of Drug Sensitivity in Cancer
TIDE: Tumor Immune Dysfunction and Exclusion
GSEA: Gene set enrichment analysis
MSigDB: The Molecular Signatures Database
C-index: Concordance index
HPA: Human Protein Atlas
IHC: Immunohistochemistry
DAB: Diaminobenzidine tetrachloride
qPCR: Real-time polymerase chain reaction
OS: Overall survival
HR: Hazard ratio
CI: Confidence interval
PDGFRA: Platelet derived growth factor receptor alpha
ZEB1: Zinc finger E-box binding homeobox 1
FOXF1: Forkhead box F1
SPARC: Secreted protein acidic and cysteine rich
MMP2: Matrix metallopeptidase 2
FN1: Fibronectin 1
HSPB1: Heat shock protein family B (small) member 1
S100A13: S100 calcium binding protein A13
PPP1R14A: Protein phosphatase 1 regulatory inhibitor subunit 14A
CSRP2: Cysteine and glycine-rich protein 2
TPM2: Tropomyosin 2
CEBPD: CCAAT/enhancer binding protein
TIMP1: Tissue inhibitor of metalloproteinases 1
SPINK1: Serine peptidase inhibitor Kazal type 1
CXCL1: C-X-C motif chemokine ligand 1
VEGF: Vascular endothelial growth factor
