# Supplementary material for: Integrated single-cell and bulk RNA sequencing analysis identifies a cancer associated fibroblast-related signature for predicting prognosis and therapeutic responses in colorectal cancer
Source: Cancer Cell Int. 2021 Oct 20;21:552. doi: 10.1186/s12935-021-02252-9 (PMC8529760; doi:10.1186/s12935-021-02252-9)
Supplement: Supplementary file 1 — Additional file 1: Table 1. qRT-PCR primer sequences. [file 12935_2021_2252_MOESM1_ESM.docx]

Supplementary Table 1. qRT-PCR primer sequences

| mRNA | Primer Sequence |
| --- | --- |
| CEBPD | Forward: GCCATGTACGACGACGAGAG  Reverse: TGTGATTGCTGTTGAAGAGGTC |
| CSRP2 | Forward: GGGCCAAAAGGCTACGGTTAT  Reverse: GCAGCATATACAGAATCCCCAC |
| CXCL1 | Forward: TCCTGCATCCCCCATAGTTA  Reverse: CTTCAGGAACAGCCACCAGT |
| HSPB1 | Forward: TGGACCCCACCCAAGTTTC  Reverse: CGGCAGTCTCATCGGATTTT |
| PPP1R14A | Forward: GACATGCCCGATGAGATCAAC  Reverse: CTCGACAGGTTTCCCACATGA |
| S100A13 | Forward: GATAGCCTCAGCGTCAACGAG  Reverse: CCTGATTCACATCCAAGCTCTT |
| SPINK1 | Forward: TCTATCTGGTAACACTGGAGCTG  Reverse: ACACGCATTCATTGGGATAAGT |
| TIMP1 | Forward: AGAGTGTCTGCGGATACTTCC  Reverse: CCAACAGTGTAGGTCTTGGTG |
| TPM2 | Forward: CTGAGACCCGAGCAGAGTTTG  Reverse: TGAATCTCGACGTTCTCCTCC |
