# Supplementary figures and images for: Integrated single-cell and bulk RNA sequencing analysis identifies a cancer associated fibroblast-related signature for predicting prognosis and therapeutic responses in colorectal cancer
Source: Cancer Cell Int. 2021 Oct 20;21:552. doi: 10.1186/s12935-021-02252-9 (PMC8529760; doi:10.1186/s12935-021-02252-9)

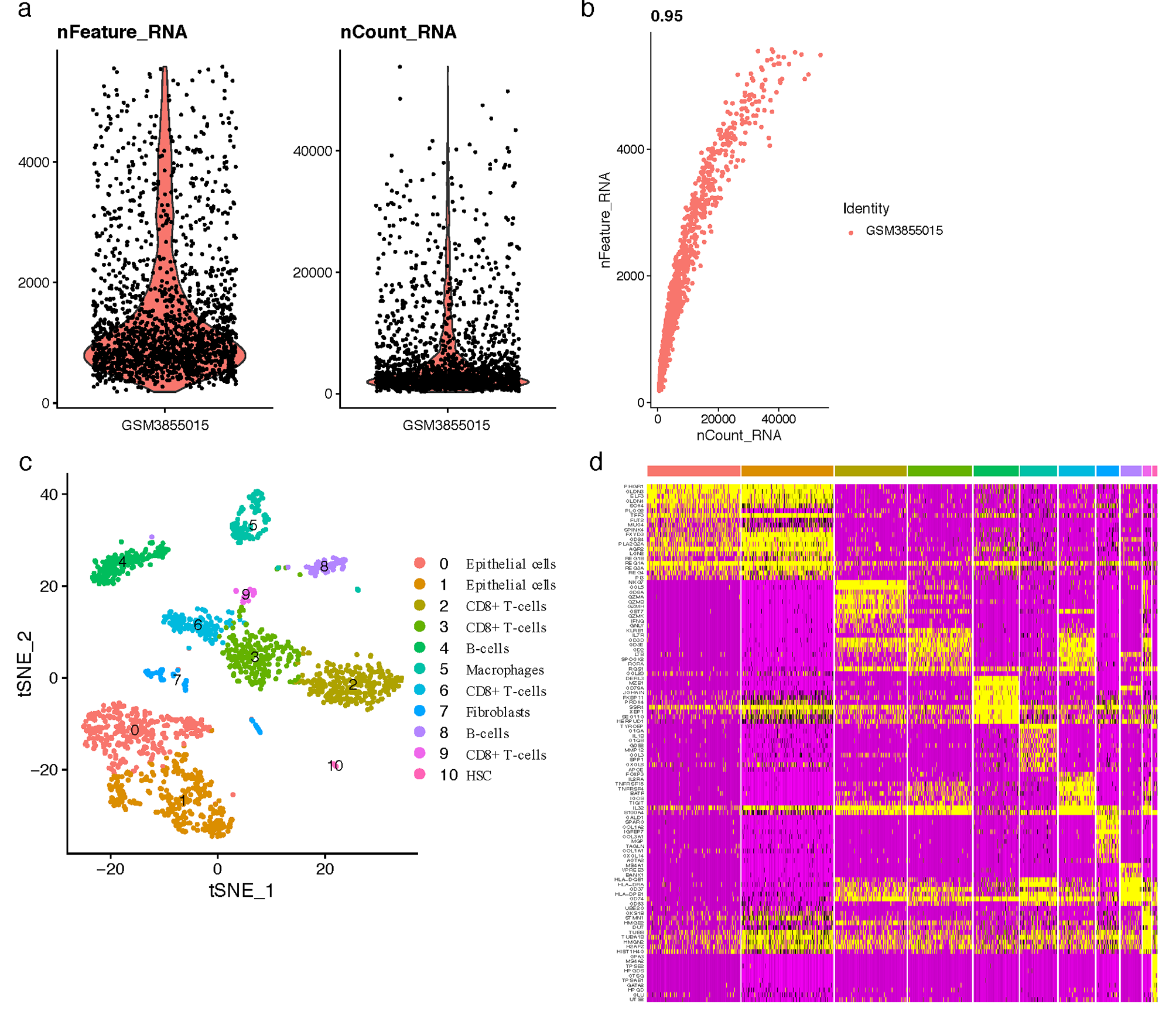

Supplement: Supplementary file 2 — Additional file 2: Figure S1. Validation analysis on single-cell RNA sequencing from 1771 cells of one CRC tissue (GSM3855015). (a) Post quality control filtering of each sequenced cell, which was plotted in violin plots to display their number of RNA features (nFeature_RNA) and absolute UMI counts (nCount_RNA). (b) Correlation analysis between nFeature and nCount. (c) Cells were clustered into 11 types via tSNE dimensionality reduction algorithm, each color represented the annotated phenotype of each cluster. (d) Heatmap depicting expressions of top 10 marker genes among 11 identified CRC cell clusters. [file 12935_2021_2252_MOESM2_ESM.tiff]

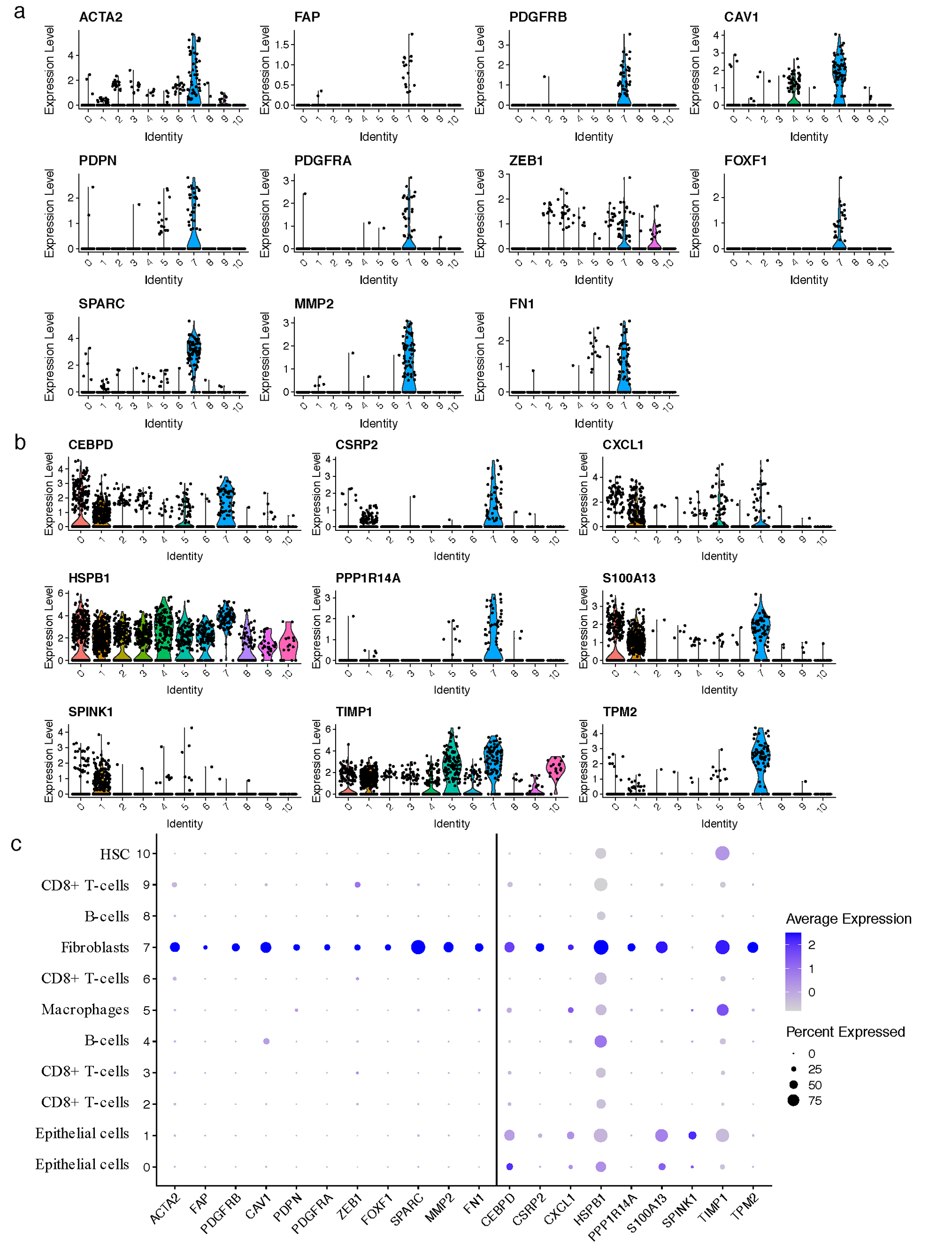

Supplement: Supplementary file 3 — Additional file 3: Figure S2. (a) Recognized and (b) the identified CAF markers expressions in single-cell clusters of GSM3855015 sample. (c) Bubble plot visualizing genes expression characteristics in single-cell RNA sequencing profile. Cell phenotypes were listed on y-axis, recognized CAF markers (left part of the dotted line) as well as the identified 9 prognostic markers (right part of the dotted line) were listed along the x-axis. Dot size reflects each gene’s expressing percentage of each cluster’s cells; dot color represents the expression level. [file 12935_2021_2252_MOESM3_ESM.tiff]
